# Indoor secondhand tobacco smoke and risk of under-five mortality in 23 sub-Saharan Africa countries: A population based study and meta-analysis

**DOI:** 10.1371/journal.pone.0177271

**Published:** 2017-05-23

**Authors:** Patrick Opiyo Owili, Miriam Adoyo Muga, Wen-Chi Pan, Hsien-Wen Kuo

**Affiliations:** 1 International Ph.D. Program in Environmental Science and Technology, Institute of Environmental and Occupational Health Sciences, School of Medicine, National Yang-Ming University, Taipei, Taiwan; 2 Institute of Community Health and Development, Great Lakes University of Kisumu, Kisumu, Kenya; 3 Institute of Environmental and Occupational Health Sciences, School of Medicine, National Yang-Ming University, Taipei, Taiwan; Liverpool School of Tropical Medicine, UNITED KINGDOM

## Abstract

**Background:**

Inhalation of secondhand smoke from tobacco results in serious health outcomes among under-five children, and yet, few studies have assessed its effect on under-five mortality. We investigated the association between frequency of exposure to household tobacco smoke and risk of under-five mortality in sub-Saharan Africa (SSA).

**Methods:**

Demographic Health Survey data of under-five children from 23 SSA countries (*n* = 787,484) were used. Cox proportional hazard models described the association between exposure to tobacco smoke and the risk of under-five mortality in each country, with age as the time-to-event indicator. Meta-analysis was used to investigate the overall effect of tobacco smoke in SSA.

**Results:**

The association between tobacco smoke exposure and the risk of under-five mortality attenuated in eight countries (Burkina Faso, Benin, Congo, Gabon, Guinea, Liberia, Togo, and Zambia) after adjustment, while the hazard ratios (HR) of daily exposure to tobacco smoke in Kenya (HR = 1.40; 95% CI, 1.16–1.70) and Namibia (HR = 1.40; 1.07–1.83) grew. The children in rural areas in SSA were 1.08 (95% CI, 1.04–1.13) times more likely to die than their urban peers. In general, the exposure to household tobacco smoke was associated with an increased risk of under-five mortality in SSA (HR = 1.09; 95% CI, 1.06–1.13).

**Conclusions:**

This study provided evidence of a positive association between exposure to household tobacco smoke and risk of under-five mortality in SSA. Policymakers in low- and middle-income countries, where tobacco control as a child health issue is relatively neglected, should integrate tobacco control measures with other child health promotion policies.

## Introduction

Exposure to tobacco smoke and the resulting inhalation of secondhand smoke (SHS) is one of the most common and extensive types of exposure in the micro-environment (i.e., indoors). Tobacco smoke’s role in negative health outcomes has been widely established in children, with links to sudden infant death syndrome (SIDS), acute respiratory infection, ear problems, intensified asthma and premature death [1–6]. However, the association between exposure to secondhand tobacco smoke and mortality of children under the age of five has yet to be adequately established [7], in part due to inadequate information on family, genetic, micro- and macro-environmental factors. These children, under the age of five, are vulnerable to indoor air pollution since most of their early years are spent indoors around their mothers or caretakers.

Globally, 40% of children are exposed to secondhand tobacco smoke, which is responsible for over 600,000 deaths per year, of which, close to a third of those deaths were children under the age of five and two-thirds were in Sub-Saharan Africa (SSA) and South Asia [8]. Moreover, exposure to indoor secondhand tobacco smoke is predominant in nations without public health regulations on 100% smoke-free environments, and these nations also account for over 90% of the global population [8]. In the year 2012, smokers numbered 1.1 billion globally, and 8 out of 10 smokers were smoking daily; though, SSA had the lowest prevalence of tobacco smoking worldwide, with the prevalence of smoking among men being just over 20% and that of women being less than 5% [9]. Global estimates indicate that 12% to 13% of children in SSA were regularly exposed to secondhand tobacco smoke [10], and yet, one study found that over 25% of the subjects were exposed secondhand tobacco smoke at home in Africa [11]. These proportions are even expected to increase as tobacco companies shift their focus to countries that do not have or are not keen to implement 100% smoke free regulations.

Our study is therefore significant in this era of the Sustainable Development Goals (SDGs), which is characterized by a heightened need to reduce the under-five mortality rate to 25 deaths or below per 1,000 live births, and a similar need to strengthen the implementation of the World Health Organization’s (WHO) Framework Convention on Tobacco Control (FCTC) in all countries [12, 13]. Also, our study not only adds to the body of knowledge on the health effects of secondhand tobacco smoke, but is the first in SSA. Hence, we aimed to determine the distribution of exposure to indoor secondhand tobacco smoke and the under-five mortality, and further explored the association between the frequency of exposure to indoor secondhand tobacco smoke and the risk of the under-five mortality in SSA. We also investigated the extent to which exposure to secondhand tobacco smoke had independent associations with under-five mortality risks in different residential areas (i.e., rural or urban).

## Methods

### Data and sample

We used the most recent cross-sectional data of 23 SSA countries collected between 2010 and 2014 by the Demographic Health Survey (DHS) for the under-five children (*n* = 787,484); and these countries included: Benin (*n* = 42,775), Bukina Faso (*n* = 53,312), Burundi (*n* = 23,209), Comoros (*n* = 9,870), Congo (*n* = 28,379), Côte d'Ivoire (*n* = 25,312), DRC (*n* = 56,391), Ethiopia (*n* = 42,849), Gabon (*n* = 17,882), Gambia (*n* = 23,980), Guinea (*n* = 25,873), Kenya (*n* = 36,947), Liberia (*n* = 27,263), Mali (n = 28,125), Mozambique (*n* = 34,766), Nigeria (*n* = 114,134), Namibia (*n* = 9,259), Rwanda (*n* = 30,597), Sierra Leone (*n* = 43,471), Togo (*n* = 25,084), Uganda (*n* = 27,748), Zambia (*n* = 43,064), and Zimbabwe (*n* = 17,194). These countries were considered because of the availability of the most recent data on indoor secondhand tobacco smoking. The DHS is a national household representative interview-based survey collected periodically in collaboration with the health ministries of these countries. The interview targets information about women of reproductive age (aged 15–49 years) and their children as well as men (aged 15–54 years). The survey targeted households and used a stratified sampling technique based on a two-stage cluster sampling design. More details on the data (e.g., sampling criteria and data processing) can be found in the final report of each specific country on the DHS program’s website [14].

### Ethics statement

The DHS program, in collaboration with the ministries of health of each country, collects data periodically for general national planning purposes. As described elsewhere [15], high ethical standards are upheld in collecting, analyzing and disseminating the DHS data. A written informed consent was also obtained from the parents of the under-five children before data collection. The survey datasets used in this study were anonymized with regard to participants’ identities. After authorization to use the dataset was obtained from the DHS, additional ethical approval to perform this particular study was obtained from the Institutional Review Board (IRB) of National Yang-Ming University.

### Measures

#### Outcome variables

We used the all-cause mortality of children between 0–59 months to define the under-five mortality (dichotomized as a binary variable) with the time-to-event being age in months. The question on mortality sought to know whether all the children born to the woman were alive or dead at the time of the interview. If a child was dead, a follow-up question was on the age of the child at death. In 2015, the estimated under-five mortality rate (deaths per 1,000 live births) of all the SSA countries in our study were higher than the global target (i.e. below 25 deaths per 1,000 live births) as follows: Benin (100), Bukina Faso (89), Burundi (82), Comoros (74), Congo (45), Côte d'Ivoire (93), DRC (98), Ethiopia (59), Gabon (51), Gambia (69), Guinea (94), Kenya (49), Liberia (70), Mali (115), Mozambique (79), Nigeria (109), Namibia (45), Rwanda (42), Sierra Leone (120), Togo (78), Uganda (55), Zambia (64), and Zimbabwe (71) [16]. However, we used the mortality information in the DHS dataset.

#### Exposure to secondhand tobacco smoke

The main independent variable was frequency of exposure to secondhand tobacco smoke in the household. The question used to obtain data for the independent variable was based on the frequency of any household member smoking inside the house which had five responses: never, daily, weekly, monthly and less than monthly. In our analysis, we combined “monthly” and “less than monthly” into one group because of the small sample size representing these categories in some countries, and hence we had four categories.

#### Other variables

Several child, parental, household and country variables were included in our study such as residence, child’s sex, breastfeeding status, number of under five children, mother’s age, education and occupation, father’s occupation, number of household members, wealth index and cooking fuel. The family’s socioeconomic status was defined by parental occupations, educations and household wealth index (a categorical variable taking one of the following values: poorest, poor, middle-class, rich, and richest). The principal component analysis (PCA) was used to construct the household wealth index from the calculated wealth scores of household assets, such as valuable goods and facilities in the household [14]. The mother’s education had four categories: no education, primary, secondary, and higher; while, maternal and paternal occupations fell into five categories: unemployed, professional/office-related, sales, farming and other services. The other variables such as mother’s age and the number of household members were however continuous variables. Child characteristic variables included the sex of the child, breastfeeding status and the number of children under the age of five in the household. Our analyses also included the administrative regions of each country (i.e., provinces) and the type of residence (i.e., urban or rural). Furthermore, the type of cooking fuel used in household was included and categorized as a clean fuel (electricity, liquid petroleum gas, natural gas, and biogas) or a pollutant fuel (kerosene, coal/lignite, charcoal, wood, straw/shrubs/grass, agricultural crop waste and dung cakes). Some of these variables have been used by authors in the study of secondhand tobacco smoke and SIDS [5].

### Statistical analysis

The proportion of children under five who were exposed to secondhand tobacco smoke and who died in each country was mapped to create a distribution in SSA. Under-five mortality and frequency of exposure to secondhand tobacco smoke were analyzed in a χ^2^ test for categorical variables and through general linear regression for continuous variables. We used Cox proportional hazards models that employ a maximum likelihood test to analyze the association between exposure to secondhand tobacco smoke and the under-five mortality, with the time-to-event variable being the age in months till the month of death. The hazard ratio (HR) for each country was produced separately after we had adjusted for other variables. To adjust for sampling design, we weighted all the analyses using complex survey method that employs Taylor series linearization by incorporating the sampling weights, country, sampling strata (cluster) and primary sampling unit.

We plotted smoothed hazard estimates to explore the change in risk of under-five mortality in SSA over the 59-month period, and to determine whether place of residence had an influence. The frequency exposure to secondhand tobacco smoke was analyzed to somewhat determine the dose-response relationship before using a binary variable (i.e., exposed vs. non-exposed) in our final analysis that investigated the overall effect of secondhand tobacco smoke on the under-five mortality in SSA by employing a random effect model. In this model, we calculated the pooled HR and its 95% confidence interval (CI) for the 23 countries with a meta-analysis technique. The estimated effect was anticipated to be heterogeneous because of the differences in the populace across and within countries in SSA, and an established approach (*I*^2^) was used to assess heterogeneity between countries’ estimates [17]. The *I*^2^ considers the additive elements resulting from the variation (heterogeneity) within the country and between the countries. We also used a funnel plot to assess bias between countries. All the Cox analyses were carried out in Stata 13.1 [18], while for mapping we used Google Earth Pro and Epi Info 7.2 [19, 20]. The meta-analysis was performed using Review Manager 5.3 [21].

## Results

### Geographical distribution of secondhand smoking and under-five mortality

**Fig 1:**
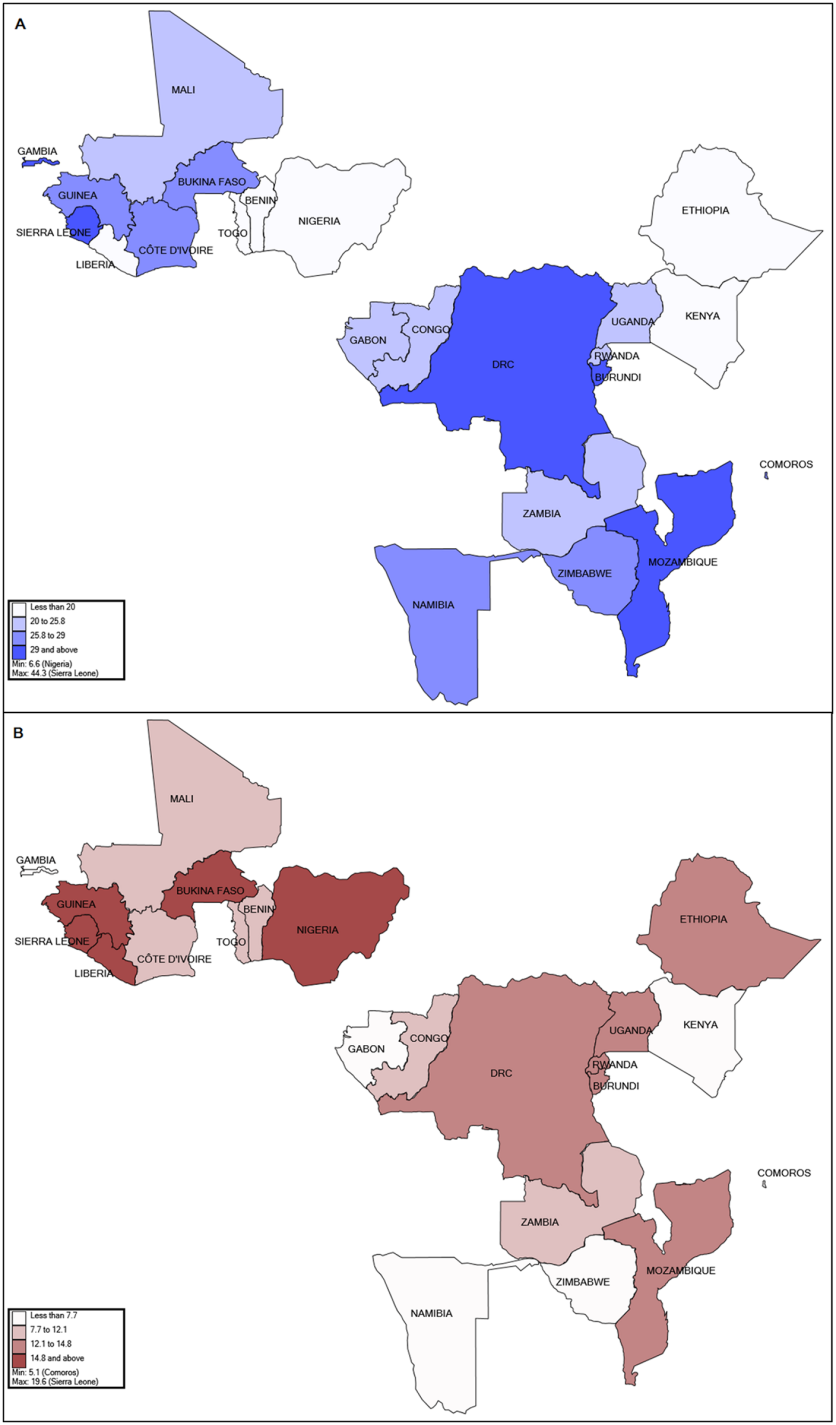
The geographical distributions for the proportions of children (A) exposed to secondhand tobacco smoke and (B) under-five mortality in the 23 sub-Saharan Africa countries.

Fig 1 shows the distribution for the proportion of secondhand tobacco smoking and under-five mortality in the 23 SSA countries. In general, some of the countries that had high proportions of secondhand smokers were also high in the under-five deaths (Sierra Leone, Guinea, Bukina Faso, DRC, Burundi, and Mozambique), while other countries were low in either the proportion of secondhand smokers (Nigeria, Ethiopia, Benin, Liberia and Togo) or in the proportion of the under-five mortality (Comoros, Namibia, Gabon, Zimbabwe, and Gambia). Only Kenya was low for both indicators.

### Demographic and socioeconomic profile

Table 1 lists the 787,484 children by mortality. A total of 98,751 under-five children died, and all the characteristics were statistically significant at *p* < 0.001. At first evaluation, compared with the children under five who were alive, the majority of those who died in the 23 countries were living in rural areas, were male, had stopped breastfeeding, were of uneducated mothers and had fathers working in farm-related jobs. Sierra Leone (19.6%) had the highest number of under-five deaths while Comoros (5.1%) had the lowest. Country, as a factor, was significant at *p* < 0.001.

**Table 1:** Characteristics of under-five children, stratified by mortality.

	Total	Alive	Dead	*p*-value
	*N*	*N* (%_wt_)^a^	*N* (%_wt_)^a^	
**All**	787,484	688,733 (87.4)	98,751 (12.6)	
**Country**, y				< 0.001
Benin, 2011/12	42,775	39,378 (92.3)	3,397 (7.7)	
Bukina Faso, 2010	53,312	44,961 (84.4)	8,351 (15.6)	
Burundi, 2010	23,209	19,996 (85.4)	3,213 (14.6)	
Comoros, 2012	9,870	9,376 (94.9)	494 (5.1)	
Congo, 2011/12	28,379	25,729 (91.6)	2,650 (8.4)	
Côte d'Ivoire, 2011/12	25,312	21,996 (88.0)	3,316 (12.0)	
DRC, 2013/14	56,391	49,389 (87.9)	7,002 (12.1)	
Ethiopia, 2011	42,849	36,589 (85.7)	6,260 (14.3)	
Gabon, 2012	17,882	16,663 (94.0)	1,219 (6.0)	
Gambia, 2013	23,980	22,223 (92.9)	1,757 (7.1)	
Guinea, 2012	25,873	22,008 (85.2)	3,865 (14.8)	
Kenya, 2014	36,947	34,415 (93.0)	2,532 (7.0)	
Liberia, 2013	27,263	22,761 (84.0)	4,502 (16.0)	
Mali, 2012/13	28,125	25,048 (88.9)	3,077 (11.1)	
Mozambique, 2011	34,766	30,282 (86.2)	4,484 (13.8)	
Namibia, 2013	9,259	8,729 (94.3)	530 (5.7)	
Nigeria, 2013	114,134	96,105 (84.2)	18,029 (15.8)	
Rwanda, 2010	30,597	26,576 (86.7)	4,021 (13.3)	
Sierra Leone, 2013	43,471	35,151 (80.4)	8,320 (19.6)	
Togo, 2013/14	25,084	22,360 (89.8)	2,724 (10.2)	
Uganda, 2011	27,748	24,404 (87.8)	3,344 (12.2)	
Zambia, 2013/14	43,064	38,508 (89.6)	4,556 (10.4)	
Zimbabwe, 2010/11	17,194	16,086 (93.2)	1,108 (6.8)	
**Residence**				< 0.001
Rural	563,543	486,806 (86.1)	76,737 (13.9)	
Urban	223,941	201,927 (90.7)	22,014 (9.3)	
*Child’s characteristics*				
**Sex**				< 0.001
Female	386,512	341,249 (88.3)	45,263 (11.7)	
Male	400,972	347,484 (86.6)	53,488 (13.4)	
**Currently breastfeeding**				< 0.001
No	497,329	428,856 (86.2)	68,473 (13.8)	
Yes	290,155	259,877 (89.5)	30,278 (10.5)	
**No. of under-5 in HH**, *mean* (SD)^b^	1.6	1.7 (1.4)	1.4 (1.3)	< 0.001
*Parent’s characteristics*				
**Mother’s age**, *mean* (SD)^b^	35.5	35.3 (7.9)	36.8 (7.7)	< 0.001
**Mother’s education**				< 0.001
No education	402,063	341,804 (84.9)	60,259 (15.1)	
Primary	245,190	217,567 (88.7)	27,623 (11.3)	
Secondary	124,872	114,882 (92.2)	9,990 (7.8)	
Higher	15,359	14,480 (94.4)	879 (5.6)	
**Mother’s occupation**				< 0.001
Not employed	198,874	176,760 (88.7)	22,114 (11.3)	
Professional/office-related	23,502	21,956 (93.4)	1,546 (6.6)	
Sales	167,067	146,127 (87.7)	20,940 (12.3)	
Farming	306,079	263,362 (85.8)	42,717 (14.2)	
Other services	91,962	80,528 (90.0)	11,434 (12.0)	
**Father’s occupation**				< 0.001
Not employed	67,156	59,610 (88.5)	7,546 (11.5)	
Professional/office-related	76,133	68,805 (90.7)	7,328 (9.3)	
Sales	66,849	58,725 (88.0)	8,124 (12.0)	
Farming	386,964	331,667 (85.5)	55,297 (14.5)	
Other services	190,382	169,926 (89.4)	20,456 (10.6)	
*Household characteristics*				
**No. of HH members**, *mean* (SD)^b^	7.3	7.4 (4.1)	7.0 (3.8)	< 0.001
**Wealth index**				< 0.001
Poorest	203,581	173,831 (84.6)	39,750 (15.4)	
Poorer	173,256	149,193 (85.8)	24,063 (14.2)	
Middle-class	157,915	137,782 (87.4)	20,133 (12.6)	
Richer	140,867	125,058 (89.0)	15,809 (11.0)	
Richest	111,865	102,869 (92.2)	8,996 (7.8)	
**Cooking fuel**				< 0.001
Clean	32,039	30,217 (94.4)	1,822 (5.6)	
Pollutant	755,400	658,471 (87.1)	96,929 (12.9)	
**Secondhand smoking in HH**				< 0.001
Never	609,598	535,362 (87.9)	74,236 (12.1)	
Daily	148,404	127,684 (85.7)	20,720 (14.3)	
Weekly	18,013	15,638 (86.4)	2,375 (13.6)	
Monthly	11,469	10,049 (88.2)	1,420 (11.8)	

^a^ The proportions were weighted (i.e. %_wt_) using Taylor series linearization by including the country, sampling weights, sampling strata (cluster) and primary sampling unit;

^b^ General linear regression was employed for continuous indicators;

*N*, Unweighted number of under-five children; HH, Household; SD, Standard Deviation.

Table 2 lists the children by secondhand smoking status. From it, we find that the majority of the children under five who were passively smoking daily were dwelling in rural areas, had uneducated mothers and had fathers who were farmers. Sierra Leone (43.4%) also had the highest proportion of under-five children who were daily exposed to indoor tobacco smoke while Nigeria (5.8%) had the lowest proportion. All the characteristics were statistically significant at *p* < 0.001, except for sex (*p* = 0.847).

**Table 2:** Characteristics of under-five children, stratified by frequency of exposure to secondhand tobacco smoke.

	Never	Monthly	Weekly	Daily	*p*-value
	*N* (%_wt_)^a^	*N* (%_wt_)^a^	*N* (%_wt_)^a^	*N* (%_wt_)^a^	
**Total *N***	609,598 (78.1)	11,469 (1.4)	18,013 (2.3)	148,404 (18.2)	
**Country**, y					< 0.001
Benin, 2011/12	36,526 (85.8)	407 (1.0)	713 (1.7)	5,129 (11.5)	
Bukina Faso, 2010	39,125 (72.7)	1,339 (2.2)	2,277 (1.7)	10,571 (20.4)	
Burundi, 2010	15,515 (65.7)	921 (3.8)	709 (3.3)	6,064 (27.2)	
Comoros, 2012	7,092 (73.5)	97 (1.0)	184 (2.0)	2,497 (23.5)	
Congo, 2011/12	21,353 (79.8)	183 (0.6)	691 (2.2)	6,152 (17.4)	
Côte d'Ivoire, 2011/12	18,352 (72.9)	307 (1.1)	768 (3.0)	5,885 (22.9)	
DRC, 2013/14	38,316 (68.5)	1,337 (2.7)	2,266 (4.3)	14,472 (24.5)	
Ethiopia, 2011	34,758 (86.6)	928 (1.5)	1,387 (3.0)	5,776 (8.9)	
Gabon, 2012	12,272 (74.4)	198 (1.6)	437 (3.0)	4,975 (21.0)	
Gambia, 2013	16,765 (71.0)	411 (1.6)	578 (2.0)	6,226 (25.4)	
Guinea, 2012	19,317 (74.2)	261 (0.9)	517 (1.9)	5,778 (23.0)	
Kenya, 2014	31,352 (84.5)	477 (1.2)	780 (2.1)	4,338 (12.2)	
Liberia, 2013	22,236 (84.5)	98 (0.2)	359 (0.7)	4,570 (14.6)	
Mali, 2012/13	22,373 (80.0)	275 (1.1)	461 (1.7)	5,016 (17.3)	
Mozambique, 2011	24,940 (70.7)	856 (1.9)	715 (1.8)	8,255 (25.6)	
Namibia, 2013	6,360 (73.3)	56 (0.8)	210 (2.4)	2,633 (23.5)	
Nigeria, 2013	106,674 (93.4)	273 (0.2)	766 (0.6)	6,421 (5.8)	
Rwanda, 2010	24,059 (78.6)	140 (0.4)	541 (1.8)	5,857 (19.2)	
Sierra Leone, 2013	25,088 (55.7)	98 (0.2)	282 (0.7)	18,003 (43.4)	
Togo, 2013/14	19,674 (80.9)	147 (0.5)	504 (2.0)	4,759 (16.6)	
Uganda, 2011	20,677 (74.3)	1,397 (5.2)	974 (3.8)	4,700 (16.7)	
Zambia, 2013/14	34,100 (79.9)	632 (1.3)	1,374 (3.2)	6,958 (15.6)	
Zimbabwe, 2010/11	12,674 (73.6)	631 (4.1)	520 (3.2)	3,369 (19.1)	
**Residence**					< 0.001
Rural	427,894 (76.2)	8,425 (1.5)	13,515 (2.5)	113,709 (19.8)	
Urban	181,704 (82.4)	3,044 (1.2)	4,498 (2.0)	34,695 (14.4)	
*Child’s characteristics*					
**Sex**					0.847
Female	299,271 (78.1)	5,647 (1.4)	8,797 (2.3)	72,797 (18.2)	
Male	310,327 (78.0)	5,822 (1.4)	9,216 (2.3)	75,607 (18.3)	
**Currently breastfeeding**					< 0.001
No	389,497 (78.9)	6,960 (1.4)	10,444 (2.1)	90,428 (17.6)	
Yes	220,101 (76.7)	4,509 (1.5)	7,569 (2.6)	57,976 (19.2)	
**No. of under-5 in HH**, *mean* (SD)^b^	1.6 (1.3)	1.7 (1.3)	1.7 (1.7)	1.7 (1.4)	< 0.001
*Parent’s characteristics*					
**Mother’s age**, *mean* (SD)^b^	35.5 (7.9)	35.1 (7.7)	34.9 (7.8)	35.5 (7.9)	< 0.001
**Mother’s education**					< 0.001
No education	306,587 (76.7)	5,373 (1.3)	8,776 (2.2)	81,327 (19.8)	
Primary	186,253 (76.8)	4,148 (1.7)	6,461 (2.6)	48,328 (18.9)	
Secondary	102,592 (82.8)	1,769 (1.5)	2,653 (2.2)	17,858 (13.5)	
Higher	14,166 (92.1)	179 (1.0)	123 (1.0)	891 (5.9)	
**Mother’s occupation**					< 0.001
Not employed	156,429 (79.9)	2,719 (1.2)	4,828 (2.4)	34,898 (16.5)	
Professional/office-related	20,411 (87.5)	321 (1.3)	335 (1.4)	2,435 (9.8)	
Sales	137,673 (83.2)	2,201 (1.2)	3,157 (2.0)	24,036 (13.6)	
Farming	221,860 (72.6)	5,029 (1.7)	7,982 (2.6)	71,208 (23.1)	
Other services	73,225 (79.8)	1,199 (1.2)	1,711 (2.0)	15,827 (17.0)	
**Father’s occupation**					< 0.001
Not employed	51,793 (77.4)	1,834 (2.8)	1,723 (2.7)	11,806 (17.1)	
Professional/office-related	64,860 (85.4)	897 (1.1)	1,337 (2.0)	9,039 (11.5)	
Sales	56,525 (84.6)	958 (1.4)	1,284 (2.0)	8,082 (12.0)	
Farming	285,686 (74.5)	5,352 (1.3)	9,748 (2.5)	86,178 (21.7)	
Other services	150,734 (80.0)	2,428 (1.2)	3,921 (2.1)	33,299 (16.7)	
*Household characteristics*					
**No. of HH members**, *mean* (SD)^b^	7.2 (3.9)	7.3 (3.9)	7.4 (5.6)	7.7 (4.5)	< 0.001
**Cooking fuel**					< 0.001
Clean	26,134 (82.5)	464 (1.5)	660 (2.4)	4,781 (13.6)	
Pollutant	583,419 (77.8)	11,005 (1.4)	17,353 (2.3)	143,623 (18.5)	
**Wealth index**					< 0.001
Poorest	150,193 (74.6)	2,704 (1.3)	4,773 (2.4)	45,911 (21.7)	
Poorer	129,963 (74.9)	2,359 (1.4)	4,432 (2.5)	36,502 (21.2)	
Middle	122,267 (77.6)	2,257 (1.4)	3,434 (2.2)	29,957 (18.8)	
Richer	113,374 (80.8)	2,380 (1.7)	3,073 (2.2)	22,040 (15.3)	
Richest	93,801 (85.0)	1,769 (1.4)	2,301 (2.0)	13,994 (11.6)	

^a^ The proportions were weighted (i.e. %_wt_) using Taylor series linearization by including the country, sampling weights, sampling strata (cluster) and primary sampling unit;

^b^ General linear regression was employed for continuous indicators;

*N*, Unweighted number of under-five children; HH, Household; SD, Standard Deviation

### Secondhand tobacco smoke and risk of under-five mortality by country

In the unadjusted models that considered secondhand smoking in country-specific analyses, daily exposure to secondhand tobacco smoke was associated with a higher risk of under-five mortality in fifteen countries (Table 3). However, after adjusting for all the variables, we found the association attenuated in eight countries (Bukina Faso, Benin, Congo, Gabon, Guinea, Liberia, Togo, and Zambia), while the associations of daily exposure to secondhand tobacco smoke and risk of under-five death in Kenya (HR = 1.40; 95% CI, 1.16–1.70) and Namibia (HR = 1.40; 1.07–1.83) became statistically significant and with the highest HRs. Moreover, after dichotomizing secondhand smoking status, we found that countries that had significant smoking-mortality associations (i.e. daily exposure) were still significant.

**Table 3:** Cox proportional hazard ratios (HRs) for under-five mortality incidence for each country.

	Frequency of exposure to secondhand tobacco smoke (Reference group: Never smoke)						Binary indicator	
	Unadjusted HR (95% CI)			Adjusted HR (95% CI)^a^			Adjusted HR (95% CI)^a^	
	Monthly	Weekly	Daily	Monthly	Weekly	Daily	Never	Secondhand smoke
**Country**								
Benin	1.15 (0.75, 1.77)	1.05 (0.73, 1.51)	1.18 (1.03, 1.37)^b^	1.15 (0.75, 1.77)	1.06 (0.81, 1.40)	1.12 (0.96, 1.30)	1	1.11 (0.97, 1.28)
Bukina Faso	0.92 (0.74, 1.13)	1.23 (1.04, 1.45)^b^	1.09 (1.01, 1.17)^b^	0.87 (0.70, 1.06)	1.07 (0.91, 1.27)	0.96 (0.89, 1.04)	1	0.97 (0.90, 1.04)
Burundi	0.97 (0.74, 1.29)	1.05 (0.81, 1.36)	1.15 (1.04, 1.27)^b^	1.10 (0.83, 1.45)	1.05 (0.85, 1.31)	1.11 (1.00, 1.24)^b^	1	1.11 (1.00, 1.22)^b^
Comoros	1.18 (0.42, 3.31)	1.18 (0.47, 2.92)	0.79 (0.52, 1.20)	0.91 (0.30, 2.72)	1.12 (0.45, 2.80)	1.03 (0.68, 1.55)	1	1.03 (0.70, 1.52)
Congo	1.22 (0.51, 2.95)	1.14 (0.75, 1.73)	1.26 (1.06, 1.49)^c^	1.08 (0.44, 2.66)	0.94 (0.62, 1.42)	1.05 (0.91, 1.21)	1	1.03 (0.90, 1.18)
Côte d'Ivoire	1.41 (0.99, 2.01)	1.06 (0.73, 1.52)	1.34 (1.16, 1.55)^d^	1.25 (0.89, 1.74)	1.02 (0.74, 1.42)	1.16 (1.01, 1.33)^b^	1	1.15 (1.01, 1.30)^b^
DRC	1.00 (0.75, 1.33)	1.09 (0.92, 1.31)	1.10 (0.98, 1.24)	0.98 (0.75, 1.29)	1.07 (0.90, 1.28)	1.08 (0.97, 1.20)	1	1.07 (0.97, 1.18)
Ethiopia	1.10 (0.81, 1.50)	1.17 (0.91, 1.51)	1.23 (1.05, 1.45)^b^	0.97 (0.71, 1.31)	1.11 (0.88, 1.42)	1.21 (1.03, 1.42)^b^	1	1.16 (1.02, 1.31)^b^
Gabon	1.44 (0.41, 5.06)	1.37 (0.52, 3.59)	1.33 (1.04, 1.69)^b^	1.56 (0.46, 5.27)	1.36 (0.54, 3.45)	1.15 (0.89, 1.48)	1	1.19 (0.91, 1.57)
Gambia	0.89 (0.52, 1.54)	1.60 (0.99, 2.57)	1.19 (0.98, 1.45)	0.68 (0.41, 1.13)	1.50 (0.89, 2.54)	1.15 (0.96, 1.37)	1	1.14 (0.96, 1.35)
Guinea	0.81 (0.60, 1.11)	1.05 (0.78, 1.41)	1.25 (1.10, 1.42)^d^	0.78 (0.57, 1.08)	1.00 (0.76, 1.32)	1.11 (0.98, 1.26)	1	1.09 (0.97, 1.22)
Kenya	0.86 (0.51, 1.46)	0.89 (0.55, 1.44)	1.20 (0.98, 1.49)	0.85 (0.50, 1.44)	0.98 (0.60, 1.60)	1.40 (1.16, 1.70)^d^	1	1.30 (1.08, 1.56)^c^
Liberia	1.00 (0.63, 1.61)	1.04 (0.80, 1.35)	1.14 (1.00, 1.29)^b^	0.65 (0.30, 1.40)	1.04 (0.79, 1.36)	1.08 (0.94, 1.24)	1	1.07 (0.93, 1.23)
Mali	0.87 (0.59, 1.29)	0.75 (0.51, 1.09)	1.04 (0.91, 1.19)	0.84 (0.57, 1.22)	0.76 (0.53, 1.08)	1.06 (0.93, 1.21)	1	1.02 (0.90, 1.15)
Mozambique	0.96 (0.72, 1.27)	1.31 (1.05, 1.65)^b^	1.42 (1.27, 1.60)^d^	1.13 (0.82, 1.56)	1.48 (1.20, 1.82)^d^	1.26 (1.13, 1.42)^d^	1	1.27 (1.14, 1.41)^d^
Namibia	1.19 (0.15, 9.44)	0.94 (0.44, 2.00)	1.23 (0.95, 1.60)	0.88 (0.11, 6.89)	0.91 (0.39, 2.14)	1.40 (1.08, 1.83)^b^	1	1.33 (1.03, 1.72)^b^
Nigeria	0.67 (0.42, 1.06)	0.77 (0.57, 1.04)	0.89 (0.78, 1.02)	0.69 (0.40, 1.20)	0.85 (0.58, 1.24)	0.97 (0.85, 1.10)	1	0.94 (0.83, 1.07)
Rwanda	1.28 (0.81, 2.05)	0.97 (0.74, 1.27)	1.14 (1.03, 1.26)^b^	1.26 (0.82, 1.93)	0.98 (0.76, 1.25)	1.13 (1.02, 1.24)^b^	1	1.12 (1.02, 1.22)^b^
Sierra Leone	0.88 (0.49, 1.57)	1.23 (0.90, 1.67)	1.20 (1.12, 1.29)^d^	1.08 (0.66, 1.77)	1.26 (0.94, 1.67)	1.14 (1.07, 1.22)^d^	1	1.14 (1.07, 1.22)^d^
Togo	1.24 (0.64, 2.40)	1.02 (0.73, 1.42)	1.28 (1.14, 1.44)^d^	0.85 (0.45, 1.60)	0.97 (0.72, 1.31)	1.08 (0.95, 1.23)	1	1.06 (0.94, 1.19)
Uganda	1.17 (0.97, 1.41)	1.50 (1.19, 1.90)^d^	1.29 (1.12, 1.49)^d^	1.03 (0.85, 1.25)	1.26 (1.01, 1.58)^b^	1.14 (1.00, 1.31)^b^	1	1.14 (1.02, 1.27)^b^
Zambia	1.29 (0.87, 1.92)	1.20 (0.93, 1.54)	1.14 (1.00, 1.29)^b^	1.24 (0.86, 1.80)	1.12 (0.89, 1.41)	1.05 (0.92, 1.19)	1	1.07 (0.95, 1.20)
Zimbabwe	1.06 (0.71, 1.58)	0.97 (0.57, 1.67)	1.00 (0.80, 1.26)	0.94 (0.63, 1.41)	0.95 (0.55, 1.63)	0.91 (0.73, 1.13)	1	0.92 (0.76, 1.11)

^a^ Adjusted for residence, sex, breastfeeding status, number of under-5 children, mother’s age, mother’s education, mother’s occupation, father’s occupation, number of household members, wealth index, and cooking fuel;

^b^ *p* < 0.05;

^c^ *p* < 0.01;

^d^ *p* < 0.001

### Risk of under-five mortality by residence

**Fig 2:**
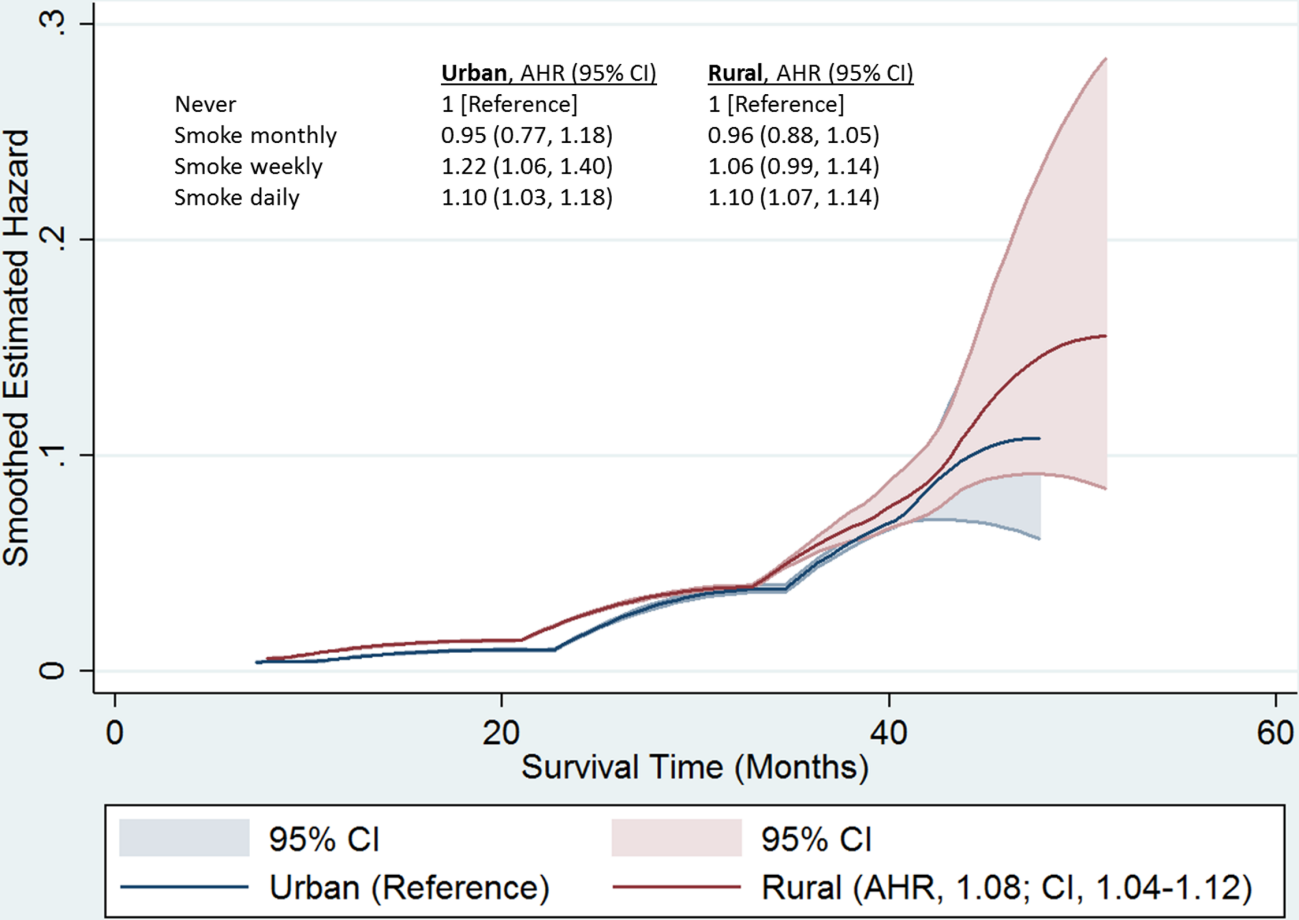
Smoothed hazard function with a 95% confidence limit for the relationship between secondhand tobacco smoke and risk of under-five mortality, by residence in sub-Saharan Africa.

Our smoothed hazard estimates indicated a pooled exposure-response relationship (Fig 2). Positive associations were observed in 20 countries, of which, nine were statistically significant (Table 3). Fig 2 indicates an urban/rural difference in the risk of the under-five mortality by exposure to secondhand tobacco smoke during early months of life, but after about 36 months the difference attenuated. However, the risk of death due to secondhand tobacco smoke increased with age in both urban and rural residences. Children under five in rural households were positively associated with the risk of under-five mortality from exposure to secondhand tobacco smoke, and had an HR of 1.08 (95% CI: 1.04–1.13) when compared to their peers in urban areas. Children under five with daily exposure to secondhand tobacco smoke in both urban and rural residences had a 10% higher risk of death than those who were not exposed.

### Overall effect of secondhand tobacco smoke on under-five mortality in SSA

**Fig 3:**
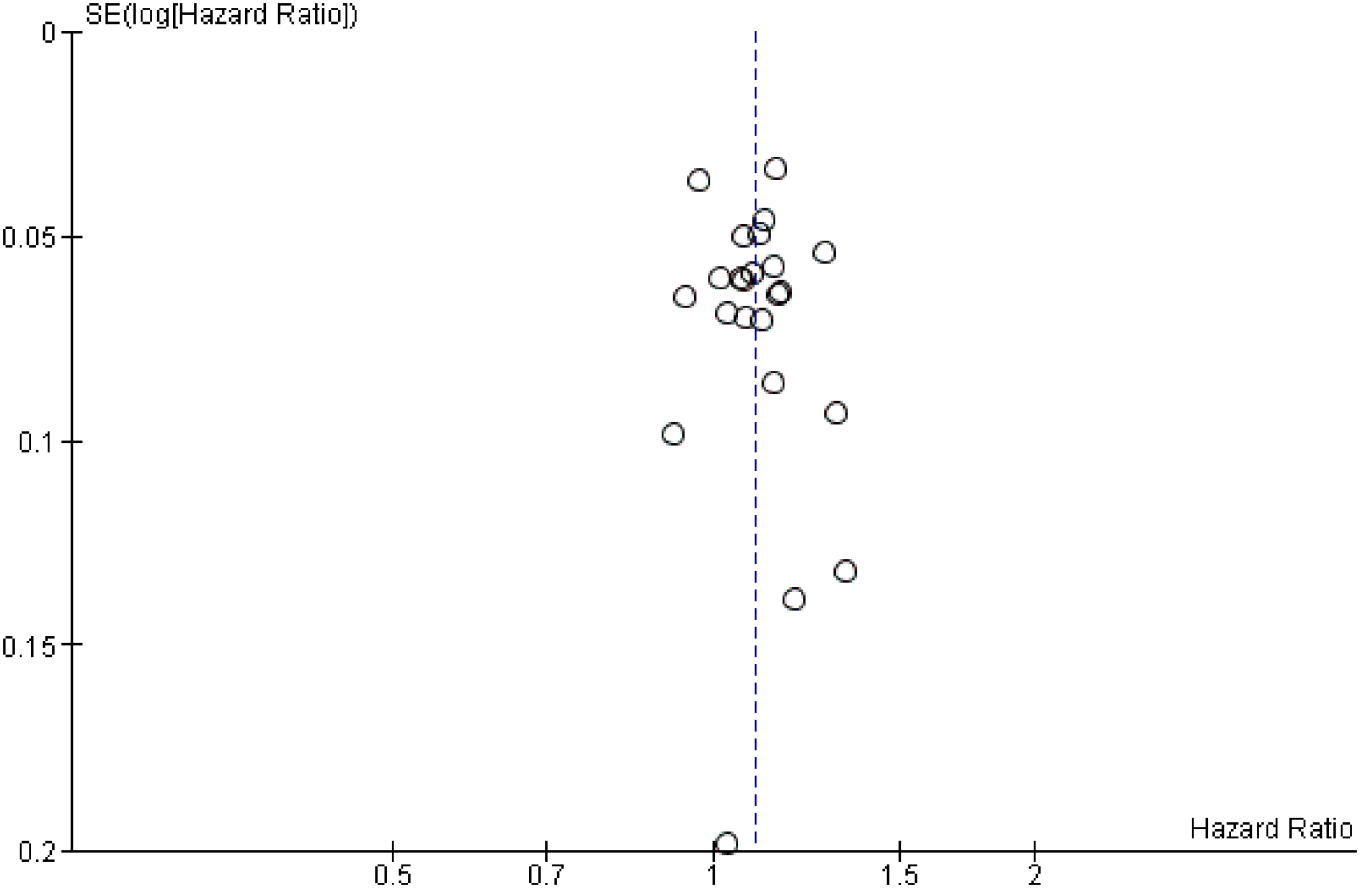
Funnel plot for exposure to secondhand tobacco smoke and risk of under-five mortality. The plot’s the x-axis is the hazard ratio for each country while the y-axis is the standard error of the hazard ratio (random effects model). The countries’ (cycles) pooled effect estimate is indicated by the dotted vertical line.

**Fig 4:**
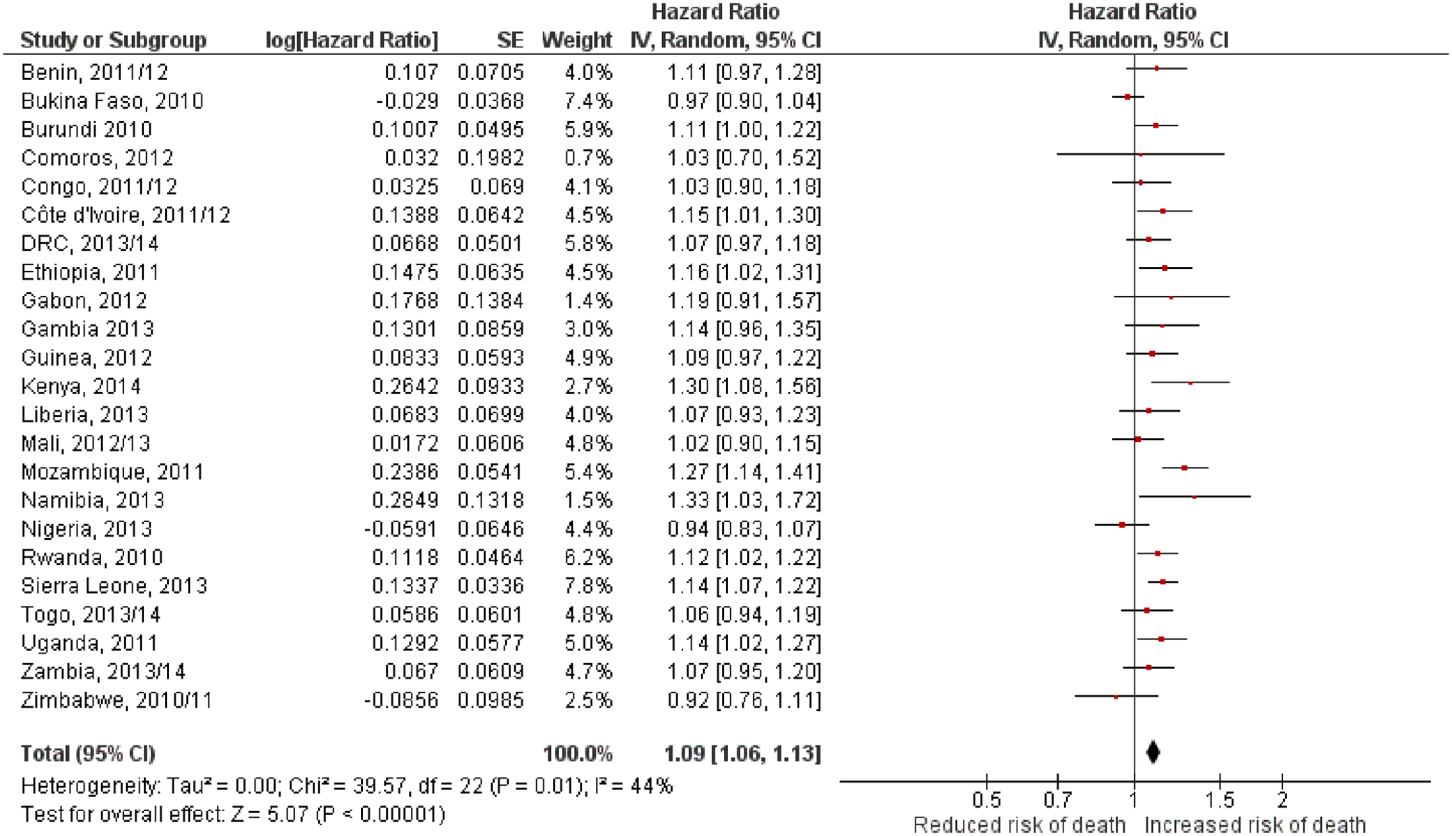
Relationship between exposure to secondhand tobacco smoke and the risk of under-five mortality in Sub-Saharan Africa from a meta-analysis of 23 countries. The red dot indicates the hazard ratio (HR) for a country. Horizontal lines cover the 95% confidence intervals (CI). The diamond is centered at the pooled HR and is as wide as the 95% CI. HR > 1 implies a higher risk of mortality for the children under five exposed to secondhand tobacco smoke.

The funnel plot for the exposure to indoor secondhand tobacco smoke and risk of under-five mortality indicates no evidence of bias (Fig 3). The funnel plot which represents a scatter plot of the estimated effect of individual countries showed that most countries appeared in the area of higher significance (HR > 1). Nevertheless, exposure to secondhand tobacco smoke was significantly and positively associated with the risk of under-five mortality in SSA, with an HR of 1.09 (95% CI, 1.06–1.13; Fig 4) at *p* < 0.001. The analysis showed a moderate level of heterogeneity (I2 = 44%, *p* = 0.01).

## Discussion

Secondhand tobacco smoke has been shown to cause several health outcomes, including premature death in children and adults who do not smoke [1, 2]. In this study, we determined the distributions of indoor secondhand smokers and the under-five mortalities in SSA, further exploring the association between the frequency of exposure to secondhand smoke and the risk of the under-five mortality. We then investigated the residential effect to find that children in rural areas (versus urban areas) were at an increased risk of death by secondhand tobacco smoke. We found that some of the countries that had high proportions of smokers also had the high proportions of under-five mortality. Moreover, we found that the children who were exposed to secondhand tobacco smoke at a daily rate were at an increased risk of death.

To the best of our knowledge, our study is the first to show that indoor secondhand tobacco smoke exposure is associated with a higher risk for under-five mortality in SSA. Household members who smoke increased the risk of under-five mortality in some countries (Burundi, Côte d'Ivoire, Ethiopia, Kenya, Mozambique, Namibia, Rwanda, Sierra Leone, and Uganda). These results held after controlling for all the other variables. The variables included household wealth status, type of residence, administrative provinces, mother’s age, parental education levels, child’s sex, breastfeeding status, number of the children under five, household members and parental occupations. All these variables were found to be individually associated with both the exposure and the under-five mortality. These results support the findings of an Indonesian study that showed paternal smoking and risk of infant as well as under-five mortality to be significantly associated [7].

Our study also found that the risk of under-five mortality as a result of indoor secondhand tobacco smoke was higher in rural areas even though its effect increased with age in both urban and rural residence. Typically, it is expected that exposure to secondhand tobacco smoke at a much younger age would more significantly impact their health than older children. Although, the younger ones might as well have limited exposure, their physical body is not strong enough as the older ones. At the same time, the period of exposure to household secondhand tobacco smoke also increases with an increase in age, and possibly leading to an increase in the likelihood of its fatal effect on the children. The majority of these children who were exposed to secondhand tobacco smoke were however in rural areas. This might explain part of the difference, in the risk of under-five mortality, between urban and rural residence; children under five living in rural settings have a significantly greater risk of death than do their urban peers. This study also supported another finding: the population-attributable risk of under-five mortality as a result of smoking is higher in rural areas than in urban areas [7].

Majority of children under five die prematurely as a result of pneumonia caused by household air pollution, of which one source is household cigarette smoke [22]. In this light, the trend of tobacco firms progressively moving their markets from high- to low-income countries is a potentially hazardous one; the populations of low-income countries are typically not conversant with the hazardous effects of tobacco, and these countries’ governments often lack robust tobacco control measures. The result is often a high number of tobacco users with limited knowledge on hazardous effect of smoking and secondhand tobacco smoke exposure [23], leading to increasingly negative health effects at the hands of secondhand tobacco smoke [24, 25]. Tobacco companies’ activities that may exacerbate threat to public health-driven policy on tobacco controls should also be discouraged—activities such as, targeting teenagers through offering free cigarettes to increase the prevalence of smoking [24, 26]. Strengthening tobacco control measures in low- and middle-income countries (LMIC) would therefore reduce the negative health effects as a result of secondhand tobacco smoke.

Our study indicated that the dose-response relationship of the risk of death from exposure to indoor tobacco smoke increased with an increase in the duration of exposure, in this case—months. Future studies needs to explore this relationship to determine the no-threshold limit of exposure to indoor tobacco smoke. Via our meta-analysis, we found that exposure to indoor secondhand tobacco smoke had a significant positive effect on the risk of under-five mortality in SSA. This finding reaffirms that secondhand tobacco smoke has a negative effect on the health and survival of children. The continued use of cigarettes in the micro-environment (e.g., households) can hinder the effective growth and development of children, obstructing them from seeing their full potential in life. However, the risk of death, as a result of secondhand tobacco smoke, decreased in three countries (Bukina Faso, Nigeria, and Zimbabwe)–though, the HRs were not statistically significant. Nevertheless, exposure to secondhand tobacco smoke increased the risk of death, overall, even with the variation within and between SSA countries.

Our study is significant to policymakers, especially in LMIC where tobacco control as a child health issue is might be relatively neglected. With accruing research evidence that smoking is associated with increased risk of negative health outcomes in children, policymakers in LMIC should integrate tobacco control measures with other child health promotion policies, especially in SSA. The evidence in this study also suggests that other stakeholders, including the departments of health, should create smoke-free environments, implement or revise laws and regulations that would discourage smoking in smoke-free zones, control media transmission of tobaccos adverts and to strengthen the FCTC, and actively promote behavioral changes in non-jurisdictional areas, promote smoking cessation programs through educational campaigns and media ads on health effects of tobacco smoke.

Naturally, our study is not without a few limitations and strengths. First, the data used in this study pulled from a retrospective cross-sectional survey remains the main limitation, for example—there is an assumption that children’s exposure to tobacco smoke was even over time using an extrapolated cross-sectional data. Moreover, mortality data were obtained retrospectively at time of the interview while the household characteristics were extrapolated for the deceased child. Therefore, we could not ascertain the true measure of causality and hence our findings should be interpreted with more caution. Future studies should consider a prospective study to determine the true causal effect of exposure to secondhand tobacco smoke. A second limitation was in the indicators we used for secondhand tobacco smoke which would not enable us to adequately determine the true exposure amount nor a true dose-response relationship. The number of cigarettes smoked and time would also help to explain our finding. Moreover, the explanation for these associations can also be as a result of the contribution of other biological factors (e.g., low birth weight and prematurity), other micro-environment health hazards (e.g., number of household members who smoke and the number of cigarettes smoked) and ambient air pollution. Third, our results might have been influenced by recall bias. Finally, a historical comprehensive health evaluation of the mothers and their children and were not available at the time of data collection. Health history may also explain the result of our finding; of which other health-related factors might have contributed to the death of children.

A major strength of our study included the large representative sample of the SSA populace which increases the generalizability of our findings to SSA. Unlike a previous study [7], we used a multi-country data to explain the association between frequency of exposure to secondhand tobacco smoke and the under-five deaths after adjusting for several variables. Secondly, the analytical technique used in our study contributed to the internal reliability of our findings. We also used meta-analysis to explain the association between secondhand tobacco smoke and the under-five mortality. Finally, use of the most recent data minimized potential bias associated with time-effect.

### Conclusion

Our study explored whether indoor secondhand tobacco smoke was associated with the risk of death in children under five. The results indicate that secondhand tobacco smoke increases the risk of under-five mortality in SSA. Furthermore, the effect of secondhand tobacco smoke on under-five deaths was higher in children living in rural areas. More research and public health promotion to educate citizens on the health risk of secondhand tobacco smoke is therefore needed.
